# *In Vitro* Antioxidant, Antibacterial, and Cytotoxic Activity and *In Vivo* Effect of *Syngonium podophyllum* and *Eichhornia crassipes* Leaf Extracts on Isoniazid Induced Oxidative Stress and Hepatic Markers

**DOI:** 10.1155/2014/459452

**Published:** 2014-08-04

**Authors:** Shashank Kumar, Ramesh Kumar, Astha Dwivedi, Abhay K. Pandey

**Affiliations:** Department of Biochemistry, University of Allahabad, Allahabad 211002, India

## Abstract

The present study reports the* in vitro* antioxidant, antibacterial, and cytotoxic potential of *Syngonium podophyllum* (SP) and *Eichhornia crassipes* (EC) leaf aqueous extracts as well as their *in vivo* effect on oxidative stress and hepatic biomarkers in isoniazid induced rats. Phytochemical screening of extracts revealed the presence of flavonoids, terpenoids, reducing sugars, alkaloids, and saponins. Phenolic content in SP and EC extracts was 5.36 ± 0.32 and 10.63 ± 0.13 mg PGE/g, respectively, while flavonoid content was 1.26 ± 0.03 and 0.51 ± 0.03 *μ*g QE/mg, respectively. EC extract exhibited comparatively better antioxidant activity as indicated by reducing power (0.197–0.775), DPPH radical scavenging potential (11%–96%), and metal ion chelating ability (42%–93%). Both the extracts provided 13%–65% protection against lipid peroxidation in rat tissue (liver, kidney, and brain) homogenate. SP and EC extracts exhibited 51% and 43% cytotoxicity against lung cancer (NCI-H322) cell line, respectively. Both extracts demonstrated considerable antibacterial activity against *Proteus vulgaris*, *Salmonella typhi*, and *Bordetella bronchiseptica*. Coadministration of *E. crassipes* extract with isoniazid in rats accounted for 46% decrease in malondialdehyde content and 21% increase in FRAP value of plasma. It also mitigated the isoniazid induced alterations in serum enzymes (SGOT, SGPT, and ALP), total bilirubin, creatinine, and hemoglobin contents. *S. podophyllum* extract was found to be hepatotoxic.

## 1. Introduction

Free radical induced oxidative damage has long been thought to be the most important cause of many diseases such as diabetes, stroke, cancer, arteriosclerosis, and cardiovascular diseases [1, 2]. Oxidative stress affects the prooxidants and antioxidants equilibrium in biological system which leads to the modification of DNA, proteins, carbohydrates, and lipids. Hydroxyl radicals, superoxide anion radicals, and singlet oxygen are the examples of free radicals that attack the unsaturated fatty acids in the biomembranes resulting in lipid peroxidation, decrease in fluidity, loss of enzymes and receptor activity, and damage to membrane proteins and ultimately leading to cell inactivation. Lipid peroxidation is also strongly associated with aging and carcinogenesis [1, 3]. Antioxidants mitigate oxidative stress, the adverse effects of free radical. It is widely accepted that antioxidants acting as radical scavengers protect the human body against free radicals that may cause pathological conditions such asthma, inflammation, neurodegeneration, Parkinson's diseases, and mongolism [2]. Plant products are rich sources of phytochemicals and have been found to possess variety of biological activities including antioxidant, cytotoxic, and hepatoprotective potential. They act as reducing agents and reverse oxidation by donating electrons and/or hydrogen ions [4, 5].

Cancer is the second leading cause of death in the world after cardiovascular diseases. Deaths arising from cancer constitute 2-3% of the annual deaths recorded worldwide and kill about 3500 million people annually all over the world [6, 7]. It is a disorder that develops due to some unusual molecular changes within the cell. These abnormalities may be due to the effects of carcinogens, such as tobacco smoke, radiation, chemicals, or infectious agents. Several chemopreventive agents are used to treat cancer, but they cause toxicity which prevents their usage and patients seek alternative complementary methods of treatment. Hence there is an urgent need for developing new approaches and drugs to prevent and/or cure this devastating disease. Plant derived natural products such as flavonoids, terpenes, and alkaloids have received considerable attention in recent years due to their diverse pharmacological properties including cytotoxic and cancer chemopreventive effects [5].

Hepatic diseases have become one of the major causes of morbidity and mortality all over world. The drug induced liver injury is one of the most common causative factors that pose a major clinical and regulatory challenge [4]. The manifestations of drug induced hepatotoxicity are highly variable, ranging from asymptomatic elevation of liver enzymes to fulminate hepatic failure. Exposure to toxic chemicals, environmental pollutants, and drugs can cause cellular injuries through metabolic activation of reactive oxygen species (ROS). Isoniazid (Laniazid, Nydrazid), also known as isonicotinylhydrazine (INH), is an organic compound used as the first-line medication in prevention and treatment of tuberculosis. INH induces hepatotoxicity by nitrogen group in its chemical structure, as it is metabolized in the liver and gets converted to an ammonia molecule, which causes hepatitis [8]. In spite of tremendous advances in modern medicine, there are hardly any reliable drugs that protect the liver from damage and/or help in regeneration of hepatic cell. Many active plant extracts are frequently utilized to treat a wide variety of clinical conditions including hepatic anomalies. Liv 52, an ayurvedic herbal product of Himalaya Drug Company, is frequently used in the treatment of liver diseases [9]. Therefore, search of effective and safe drugs for liver disorders continues to be an area of interest.

Infectious diseases are another major problem worldwide. Synthetic antibacterial drugs are not only expensive and inadequate but are also often with side effects. The multidrug resistant microbial strains are continuously increasing. Plant secondary metabolites have beneficial medicinal effects on humans due to their interaction with potential target sites [10]. Many plant products, namely, cinnamon, clove, jambolan, pomegranate, thyme, and lantana extracts have been shown to inhibit the growth of multidrug resistant* Pseudomonas aeruginosa*. There is a need to search for new antimicrobial substances from natural sources and to develop new infection-fighting strategies to control microbial infections [11, 12].

*Syngonium podophyllum* (Araceae) a parasitic vine has large leaves in the adult form and is deeply lobed.* S. podophyllum* leaf is used against sore, dry skin, fungal infection, itching, rashes, and bruises. Leaves and bark of the plant are traditionally employed in the local folk medicine of Belize for their wound healing properties [13, 14].* Eichhornia crassipes* (Pontederiaceae), commonly known as “Common Water Hyacinth,” is a floating waterweed. Methanol extract of* E. crassipes* leaf possesses anticoagulant activity due to presence of polysaccharides, which act on the intrinsic pathway of the coagulation cascade [15]. Crude methanolic extract also shows potent activity against bacteria, fungi, and algae. Alkaloids and phthalate derivatives have been identified as antimicrobial agents in* E. crassipes* leaf methanolic extract [16]. Literature is silent about biochemical activities of* S. podophyllum* and* E. crassipes* leaves. Hence the present study was conducted to evaluate the antioxidant, antibacterial, and cytotoxic potential as well as their effect on hepatic biomarkers in isoniazid induced rats.

## 2. Methods

### 2.1. Plant Material and Preparation of Extracts

*Syngonium podophyllum* (SP) and* Eichhornia crassipes* (EC) leaves were collected during summer from the campus of University of Allahabad, India. Identification of the plant was confirmed by experts in the Department of Botany, University of Allahabad. The shade dried samples were ground into powder and extracted with water (AQ) in a Soxhlet apparatus for 6–8 h [10, 17] and lyophilized. The residues were dissolved in DMSO for determination of biological activities.

### 2.2. Phytochemical Screening

Identification of the phytoconstituents, namely, tannins, flavonoids, terpenoids, cardiac glycosides, anthraquinones, reducing sugars, alkaloids, phlobatannins, and saponins in SP and EC leaf extracts was done using standard protocols [18].

### 2.3. Determination of Total Phenolics

Total phenolic content in extracts was determined according to the protocol [19] with some modifications [10]. Modification included dissolution of extracts in DMSO instead of water. Small amount (0.2 mL) of sample (2 mg/mL in DMSO) was diluted to 3 mL with water. Twofold diluted FCR (0.5 mL) was added and the contents were mixed. After 3 min, 2 mL of 20% sodium carbonate solution was added and the tubes were placed in boiling water bath for one min followed by cooling. The absorbance was measured at 650 nm against a reagent blank using spectrophotometer (Visiscan 167, Systronics). The concentration of phenol in the test samples was expressed as mg propyl gallate equivalents/g sample (mg PGE/g). The estimation was performed in triplicate, and the results were expressed as mean ± SD.

### 2.4. Quantitative Determination of Total Flavonoid Content

Aluminum chloride colorimetric method [20] as modified by us [21] was used for determination of flavonoid content in the extracts. Small amount (0.2 mL) of extract in DMSO (2 mg/mL) was taken followed by addition of methanol (1.8 mL), 10% aluminum chloride (0.1 mL), 1 M potassium acetate (0.1 mL), and distilled water (2.8 mL). Contents were mixed and incubated at room temperature for 30 min, and then absorbance was measured at 415 nm. Calibration curve was prepared with quercetin and the amount of flavonoids in the test samples was expressed as *μ*g quercetin equivalent/mg sample (*μ*g QE/mg). Experiments were performed in triplicate and the results were expressed as mean ± SEM.

### 2.5. Reducing Power Assay

The reducing power of test extracts was determined by the methods of Oyaizu [22] with slight modifications [23]. One mL aliquots of extracts (0.025–5.0 mg/mL) prepared in DMSO were taken. To each test tube 2.5 mL of phosphate buffer (0.2 M, pH 6.6) and 2.5 mL of 1% potassium hexacyanoferrate [K_3_Fe(CN)_6_] were added and contents were mixed. Tubes were incubated at 50°C in a water bath for 20 min. The reaction was stopped by adding 2.5 mL of 10% TCA and then centrifuged at 4000 g for 10 min. One mL of the supernatant was mixed with 1 mL of distilled water and 0.5 mL of FeCl_3_ solution (0.1%, w/v) and kept at 25°C for 2 min. The absorbance was measured at 700 nm. The ascorbic acid was used as positive control. All the tests were run in triplicate and results were reported as mean ± SD.

### 2.6. Metal Ion Chelating Activity

The chelation of ferrous ions by the extracts was estimated by the method of Dinis et al. [24] as modified by us [21]. Briefly, samples (200 *μ*L) prepared in DMSO containing 0.2–1.0 mg extracts were taken and the volume was raised to 1 mL with methanol. Further 3.7 mL methanol followed by 50 *μ*L of FeCl_2_ (2 mM) was added. The reaction was initiated by the addition of 5 mM ferrozine (0.2 mL) and the mixture was shaken vigorously and left standing at room temperature for 10 min. Absorbance of the pink violet solution was then measured spectrophotometrically (Elico UV-Vis SL 164) at 562 nm. The inhibition percentage of ferrozine-Fe^2+^ complex formation was calculated by the formula given below:

(1) $$\begin{array}{c} \%\text{metal}\;\;\text{ion}\;\;\text{chelating}\;\;\text{ability}=\left[\frac{\left(A_{0}-A_{1}\right)}{A_{0}}\right]\times 100, \end{array}$$

where *A*_0_ is the absorbance of control and *A*_1_ is absorbance in the presence of the sample/standard compounds. The results were expressed as mean ± SD of three replicates.

### 2.7. DPPH Radical Scavenging Activity

The free radical scavenging activity of the extracts was measured* in vitro* by 2,2-diphenyl-1-picrylhydrazyl (DPPH) assay [19] as modified by us [10]. DMSO was used as solvent for dissolving extracts instead of methanol. Three milliliters of 0.1 mM DPPH solution prepared in methanol was added to 1 mL of the test extracts (0.025–3.0 mg/mL) dissolved in DMSO. The content was mixed and allowed to stand at room temperature for 30 min in the dark. The reduction of DPPH free radical was measured by recording the absorbance at 517 nm. The percentage scavenging activities (%Inhibition) at different concentrations of the extracts were calculated using the following formula:

(2) $$\begin{array}{c} \left(\%\right)I=\left[\frac{\left(A_{C}-A_{S}\right)}{A_{C}}\right]\times 100, \end{array}$$

where *I* is inhibition and *A*_*C*_ and *A*_*S*_ are the absorbance values of the control and the sample, respectively. Three replicates were made for each sample and results were expressed as mean ± SD.

### 2.8. Lipid Peroxidation Inhibition (LPOI) Assay

The lipo-protective efficacy of extracts was estimated by the method of Halliwell and Gutteridge [25] using some modification [26]. The tissues (liver, brain, and kidney) were isolated from normal albino Wistar rats and 10%(w/v) homogenate was prepared in phosphate buffer (0.1 M, pH 7.4 having 0.15 M KCl) using homogenizer at 4°C. The homogenate was centrifuged at 800 g for 15 min and clear cell-free supernatant was used for the study of* in vitro* lipid peroxidation. One hundred microlitre samples (containing 200 *μ*g extracts) prepared in water were taken in test tubes and evaporated to dryness. Residues were reconstituted in 1 mL KCl (0.15 M) followed by addition of tissue homogenate (0.5 mL). Peroxidation was initiated by adding 100 *μ*L FeCl_3_ (0.2 mM). After incubation at 37°C for 30 min, lipid peroxidation was monitored by the formation of thiobarbituric acid reactive substances which were estimated by adding 2 mL of ice-cold hydrochloric acid (0.25 N) containing 15% TCA, 0.38% TBA, and 0.5% BHT. The reaction mixture was incubated at 80°C for 1 h followed by cooling and centrifugation. Absorbance of the pink supernatant was measured at 532 nm. BHA was used as standard for comparison. All analyses were carried out in triplicate and results were expressed as mean ± SD. The protective effect of different extracts against lipid peroxidation (%LPOI) was calculated by using the following formula:

(3) $$\begin{array}{c} \%\text{LPOI}=\left[\frac{\left(A_{0}-A_{1}\right)}{A_{0}}\right]\times 100, \end{array}$$

where *A*_0_ is the absorbance of control and *A*_1_ is absorbance in the presence of the sample/standard compounds. The results were expressed as mean ± SD of three replicates.

### 2.9. Cytotoxic Assay by Sulforhodamine B Dye (SRB Assay)

Human cancer cell lines, namely, breast (T47D), prostate (PC3), and lung cancer (NCI-H322 and A549) cell lines, were grown and maintained in RPMI-1640 medium, pH 7.4 with 10% FCS, 100 units/mL penicillin, 100 *μ*g/mL streptomycin, and 2 mM glutamine. Cells were grown in CO_2_ incubator (Heraeus, GmbH Germany) at 37°C in the presence of 90% humidity and 5% CO_2_. The* in vitro* cytotoxicity of extracts was determined by sulforhodamine B (SRB) assay [27]. Cell suspension (100 *μ*L, 1 × 10^5^ to 2 × 10^5^ cells per mL depending upon mass doubling time of cells) was grown in 96-well tissue culture plate and incubated for 24 hours. Stock solutions of test extracts were prepared in DMSO and serially diluted with growth medium to obtain desired concentrations. 100 *μ*L test extract (100 *μ*g/well) was then added to the wells and cells were further incubated for another 48 h. The cell growth was arrested by layering 50 *μ*L of 50% TCA and incubated at 4°C for an hour followed by washing with distilled water and then air-dried. SRB (100 *μ*L, 0.4% in 1% acetic acid) was added to each well, and plates were incubated at room temperature for 30 min. The unbound SRB dye was washed with 1% acetic acid and then plates were air-dried. Tris-HCl buffer (100 *μ*L, 0.01 M, pH 10.4) was added and the absorbance was recorded on ELISA reader at 540 nm. Each test was done in triplicate. The values are reported as mean ± SD of three replicates.

### 2.10. Evaluation of Antimicrobial Activity

Antimicrobial activity of plant extracts against Gram-positive* Bacillus cereus* (MTCC 6840),* Streptococcus mutans* (MTCC 497), and Gram-negative* Proteus vulgaris* (MTCC 7299),* Salmonella typhi* (MTCC 3917), and* Bordetella bronchiseptica* (MTCC 6838) was determined using Kirby-Bauer disc diffusion method [28]. The inoculum suspension of bacterial strains was swabbed on the entire surface of Mueller-Hinton agar (MHA). Sterile 6 mm diameter paper discs (Himedia) saturated with 20 *μ*L of extracts prepared in DMSO (containing 2 mg extract/disc) were aseptically placed on the upper layer of the inoculated MHA surfaces and plates were incubated at 37°C for 24 hours. Antibacterial activity was determined by measuring diameter of the zone of inhibition (ZOI) surrounding discs. Standard antibiotic discs of meropenem (10 *μ*g/disc) and ampicillin (10 *μ*g/disc) were used as positive control. Discs containing 20 *μ*L DMSO were used as a negative control. Antimicrobial assay was performed in triplicate and results are reported as average of three replicates.

### 2.11. Assessment of Oxidative Stress and Hepatic Markers in Isoniazid Induced Rats

#### 2.11.1. Animal Model and* In Vivo* Experimental Protocol

Wistar rats weighing 150–180 g of either sex were procured from CDRI, Lucknow. They were kept in departmental animal house in well cross (23 ± 2°C) with light and dark cycles of 12 h of 1 week before and during experiments. Animals were provided with standard rodent pellet diet (Amrut, India) and water was given* ad libitum*. The* in vivo* study was performed in accordance with the Guide for the Care and Use of Laboratory Animals, as adopted and promulgated by the Institutional Animal Care Committee, CPCSEA, India. The rats were divided into five groups (Groups I–V) with six in each group. Group I received saline and served as healthy control. Hepatotoxicity was induced by the oral administration of isoniazid (75 mg/kg body weight in saline) for 10 days in Groups II–V. Liv-52 (Himalayan Drug Company, India), the known hepatoprotective drug, was administered daily in group III at a dose of 50 mg/kg body weight. Extract treated groups, that is, Group IV (*S. podophyllum*) and Group V (*E. crassipes*), received leaf extracts at dose of 400 mg/kg body weight. After the experimental period (10 days), all animals were sacrificed and the blood was collected for evaluation of biochemical parameters.

#### 2.11.2. Collection of Blood, Preparation of Hemolysate, and Isolation of Serum

Heart was punctured and 5 mL of blood was drawn. The blood (2.5 mL) was allowed to clot and serum was separated at 2500 rpm for 15 min. Remaining blood (2.5 mL) was collected into Acid-Citrate-Dextrose (ACD) vials and kept on ice for not more than 1 hour before processing. The samples were centrifuged at 3000 rpm for 15 min, plasma was collected, and red blood cells (RBCs) were washed three times with normal saline. RBC was used to prepare 1 : 20 hemolysate. Packed RBCs obtained were suspended in approximately 1 volume of 0.154 M NaCl. To 0.2 mL of this suspension, 1.8 mL of *β*-mercaptoethanol-EDTA stabilizing solution (0.05 mL of *β*-mercaptoethanol and 10 mL of neutralized 10% EDTA in 1 liter distilled water) was added. Plasma and 1 : 20 hemolysate were transferred into separate eppendorf tubes and stored at −70°C, until analysis.

#### 2.11.3. Measurement of Total Antioxidant Activity (Ferric Reducing Ability of Plasma-FRAP Value)

To estimate FRAP [29], 40 *μ*L plasma was allowed to react with 2 mL of working FRAP solution containing acetate buffer (pH 3.6), 10 mM 2,4,6-tripyridyl-s-triazine (TPTZ) in 40 mM HCl, and 20 mM FeCl_3_ *·*6H_2_O in the ratio of 10 : 1 : 1 at 37°C. Fe^+2^-TPTZ complex was measured in a UV-Vis double beam spectrophotometer at 593 nm, and time scanning was done at 30-second intervals for 4 minutes. Aqueous solution of ferrous sulfate in the range of 100–1000 *μ*mol/L was used for calibration. Using the regression equation the FRAP values (*μ*mol Fe (II)/L) of the plasma were calculated.

#### 2.11.4. Measurement of Lipid Peroxidation

Malondialdehyde (MDA) was taken as the index for lipid peroxidation and estimated separately in plasma as well as in hemolysate, by the thiobarbituric acid (TBA) method [30]. Hemolysate (0.3 mL) was mixed with 0.7 mL of 0.1 M phosphate buffer (pH 7.4) and 2 mL of TBA-TCA-HCl reagent containing 15% (w/v) TCA, 0.375% (w/v) TBA, and 0.25 N HCl in the ratio of 1 : 1 : 1, incubated in boiling water bath for 30 minutes and centrifuged to obtain clear supernatant. The absorbance of the supernatant was measured at 534 nm in a UV-Vis double beam spectrophotometer. MDA was expressed as nm per g of hemoglobin for hemolysate.

#### 2.11.5. Effect of Extracts on Serum Hepatic Markers

The biochemical parameters, namely, alkaline phosphatase (ALP, U/L), serum glutamic oxaloacetic transaminase (SGOT, U/L), serum glutamate pyruvate transaminase (SGPT, U/L), bilirubin (total and direct), and creatinine, were assayed by using commercially available kits (Erba Diagnostic Kits, Germany). Haemoglobin content was measured in whole blood by cyanmethemoglobin method [31].

### 2.12. Statistical Analysis

All experiments were carried out in triplicate. Results were expressed as mean ± standard error of mean (SEM) and mean ± standard deviation (SD). The plots were prepared using Graphpad Prism software. One-way and two-way ANOVA were used for statistical analysis. *P* values of less than 0.05 were considered significant. One-way ANOVA followed by Dunnett's Multiple Comparison Test was used to compare control group from other experimental groups.

## 3. Results

### 3.1. Phytochemical Analysis and Quantification of Total Phenolic and Flavonoid Contents

**Figure 1:**
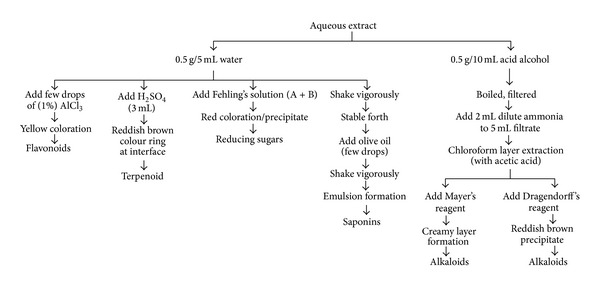
Phytochemical profile analysis of plant extracts.

Results of phytochemical screening of SP and EC leaf extracts (Figure 1) revealed the presence of flavonoids, terpenoids, reducing sugars, alkaloids, and saponins while cardiac glycosides, anthraquinone, and phlobatannins were absent in both test extracts. Total phenolic content in* S. podophyllum* and* E. crassipes* AQ extracts was 5.36 ± 0.32 and 10.63 ± 0.13 mg PGE/g sample while flavonoid content was 1.26 ± 0.03 and 0.51 ± 0.03 *μ*g QE/mg sample.

### 3.2. Reducing Power of Extracts

**Figure 2:**
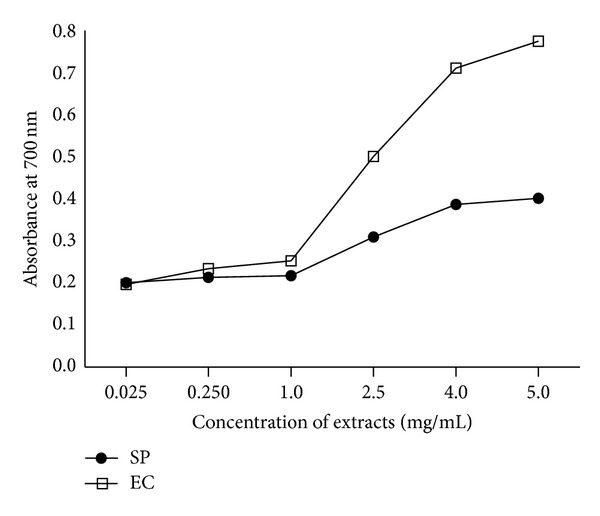
Reducing power of* S. podophyllum* (SP) and* E. crassipes* (EC) leaf extracts. Extracts were prepared in water as described in Section 2. Reducing power was measured at different concentration of extracts (0.025–5.0 mg/mL). The results are expressed as mean ± SD of three replicates (*P* < 0.05).

Reducing power of the extracts was determined at different concentrations (0.025–5.0 mg/mL). Dose dependent reducing ability was observed in test extracts at higher concentrations. Comparatively better activity was observed in EC extract than SP (Figure 2).

### 3.3. DPPH Radical Scavenging Activity

**Figure 3:**
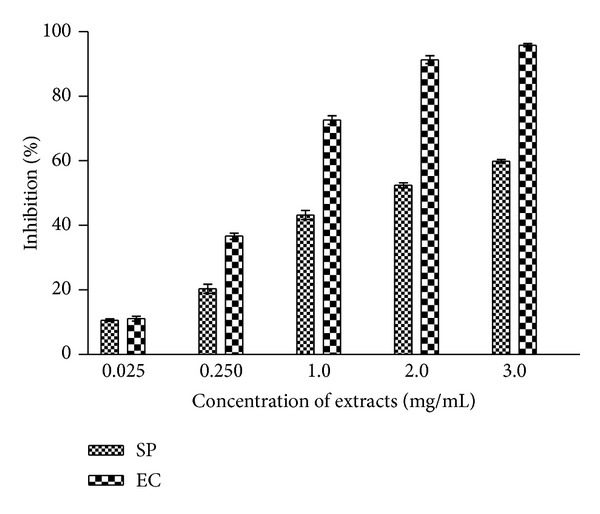
DPPH free radical scavenging activity of* S. podophyllum* and* E. crassipes* leaf extracts. Aqueous extracts were prepared as described in Section 2. Radical scavenging activity was measured at different concentration of extracts (0.025–3.0 mg/mL). The results are expressed as mean ± SD of three replicates (*P* < 0.05).

Free radical scavenging potential of test extracts at different concentrations (0.025–3.0 mg/mL) was measured by the DPPH radical scavenging assay and the results are shown in Figure 3. The degree of discoloration indicates the scavenging potentials of the extracts. Considerable scavenging potential (72–96%) in EC extract was found at higher concentrations (1–3 gm/mL). SP extract exhibited comparatively lower radical scavenging activity (11–60%) at test concentrations.

### 3.4. Metal Ion Chelating Ability

**Figure 4:**
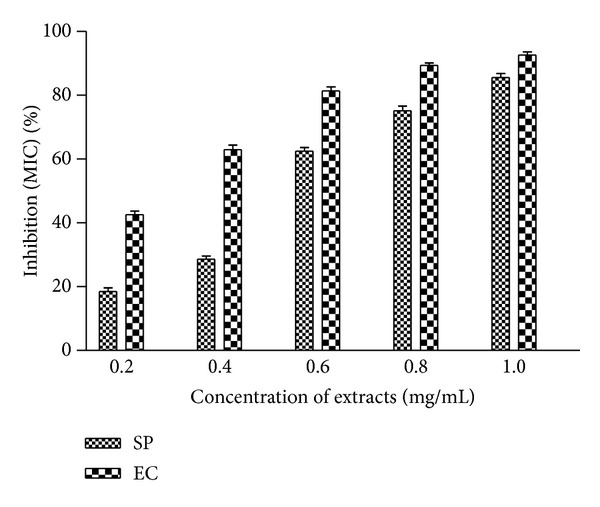
Metal ion chelating activity of* S. podophyllum* and* E. crassipes* leaf extracts. Metal ion chelating activity was measured at different concentration of extracts (0.2–1.0 mg/mL). The results are expressed as mean ± SD of three replicates (*P* < 0.05).

Metal ion chelating potential of EC and SP extracts was determined in the concentration range 0.2–1.0 mg/mL and results are depicted in Figure 4. Dose dependent chelation potential was observed in both extracts. More than 60% activity was observed in both samples at most of the test concentrations. At the highest test concentration* S. podophyllum* and* E. crassipes* extracts demonstrated 85% and 93% chelating activities, respectively.

### 3.5. Lipid Peroxidation Inhibition Activity (%LPOI)

**Figure 5:**
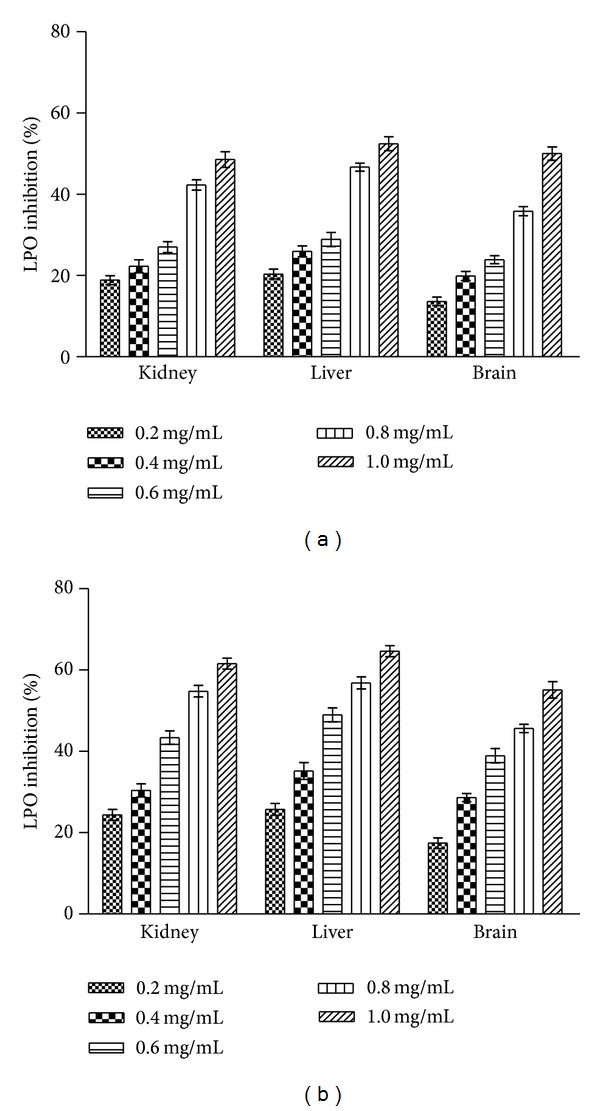
Lipoprotective efficacy of (a)* S. podophyllum* leaf and (b)* E. crassipes* leaf aqueous extracts in rat tissue homogenate. Percentage LPOI activity of extract at different concentrations (0.2–1.0 mg/mL) was assessed as an indicator to protect peroxidative damage of membrane lipids in rat tissue homogenates. The results are expressed as mean ± SD of three replicates (*P* < 0.05).

Membrane protective activity of EC and SP extracts in rat tissue (kidney, liver, and brain) homogenate was assayed at 0.2–1.0 mg/mL concentration. Dose dependent %LPOI was observed with the test extracts and results are shown in Figures 5(a) and 5(b).* E. crassipes* extract provided comparatively better protection (55–65%) against lipid peroxidation than* S. podophyllum* extract (50–52%) in liver tissue.

### 3.6. Cytotoxic Activity

**Figure 6:**
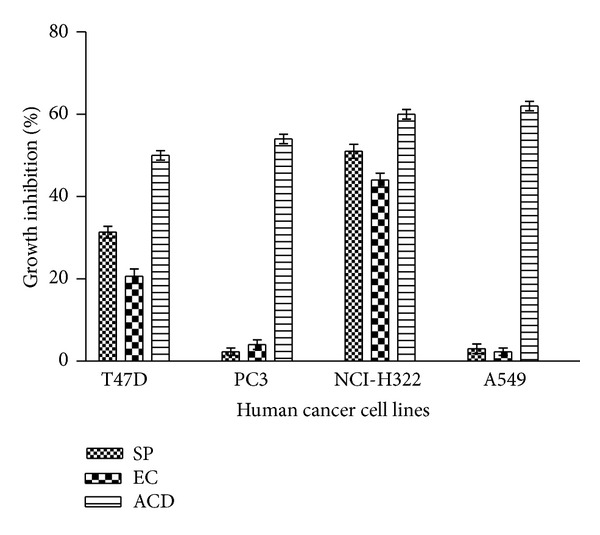
Cytotoxic effect of* S. podophyllum* and* E. crassipes* leaf aqueous extracts against cancer cell lines using SRB assay. Percentage growth inhibition of T47D (breast), PC3 (prostate), NCI-H322 (lung), and A549 (lung) cancer cell lines was assayed at 100 *μ*g/mL concentration of extracts as described in Section 2. SP:* S. podophyllum,* EC:* E. crassipes*, and ACD: anticancer drugs (mitomycin-C (10 *μ*M) against breast and prostate and 5-Flurouracil (20 *μ*M) against lung cancer cell lines). Data represent mean ± SD of three replicates (*P* < 0.05).

Anticancer activity of* S. podophyllum* and* E. crassipes* leaf extracts against T47D, PC3, NCI-H322, and A549 cell lines is shown in Figure 6. AQ fraction of SP and EC leaf exhibited 51% and 44% cytotoxic potential against NCI-H322 cell line, respectively. Against T47D cell line both samples showed 20–31% cytotoxic activity. PC3 and A549 cell lines exhibited resistance to test extracts. Standard anticancer drugs at different concentrations demonstrated 50–62% cytotoxic activity at different test cell lines (Figure 5).

### 3.7. Antibacterial Activity

The antibacterial activities of* S. podophyllum* and* E. crassipes* leaf aqueous extracts were evaluated against Gram-positive and Gram-negative bacteria (Table 1). Considerable antibacterial activity (ZOI 18–22 mm) against Gram-negative bacteria was observed in both extracts. However* B. bronchiseptica* (MTCC 6838) was resistant to antibiotics and* E. crassipes* extract. Gram-positive bacteria exhibited resistance to the extracts.

### 3.8. Total Antioxidant Capacity By FRAP Assay

**Figure 7:**
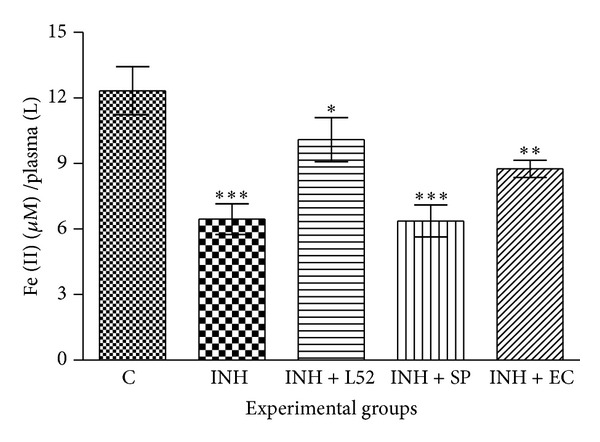
Effect of* S. podophyllum* and* E. crassipes* leaf aqueous extracts on total antioxidant capacity of plasma (measured as FRAP value) in isoniazid induced hepatotoxicity in Wistar rats. FRAP value is expressed as *μ*mol Fe (II)/L plasma. Data represent mean ± SD of three replicates (*P* < 0.05). One-way ANOVA followed by Dunnett's Multiple Comparison Test was used to compare control group from other experimental groups (*P < 0.01, **P < 0.001, and ***P < 0.0001). C: Control, INH: isoniazid, L52: Liv52, SP:* Syngonium podophyllum,* and EC:* Eichhornia crassipes.*

Isoniazid treated rats showed a decrease in FRAP content (6.25 *μ*mol Fe (II)/L plasma) compared with control group (12.38 *μ*mol Fe (II)/L plasma).* E. crassipes* leaf aqueous extract (at 400 mg/kg body weight) treated group showed increase in total antioxidant capacity of plasma (8.96 *μ*mol Fe (II)/L plasma) content in Wistar rats (Figure 7). Liv-52 treatment showed the appreciable mitigation of isoniazid induced decrease in FRAP content.* S. podophyllum* combined with isoniazid did not show any improvement in total antioxidant capacity (6.12 *μ*mol Fe (II)/L plasma).

### 3.9. Measurement of MDA Content

**Figure 8:**
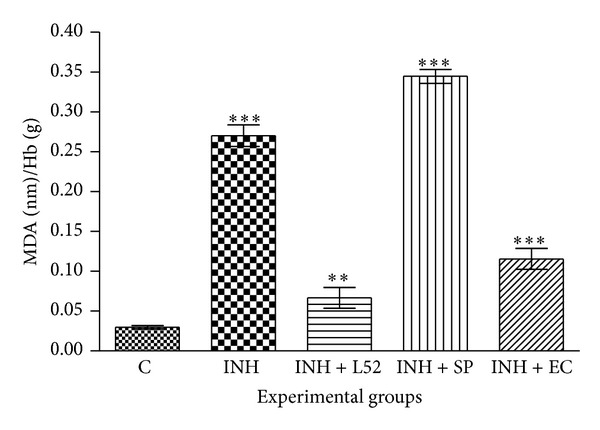
Effect of* S. podophyllum* and* E. crassipes* leaf aqueous extract on erythrocyte malondialdehyde (MDA) content in isoniazid induced hepatotoxicity in Wistar rats. Concentration of MDA is expressed as nmol/g Hb. Data represent mean ± SD of three replicates (*P* < 0.05). One-way ANOVA followed by Dunnett's Multiple Comparison Test was used to compare control group from other experimental groups (**P < 0.001 and ***P < 0.0001). C: Control, INH: isoniazid, L52, Liv52, SP:* Syngonium podophyllum,* and EC:* Eichhornia crassipes.*

Results for MDA estimation showed increase in lipid peroxidation in isoniazid and* S. podophyllum* leaf aqueous extract treated rats (0.27 and 0.34 nM/g Hb, resp.) compared with control group (0.03 nM/g Hb).* E. crassipes* leaf aqueous extract (at 400 mg/kg body weight) treated group showed decrease in MDA content (0.12 nM/g Hb). Liv-52 treatment exhibited considerable mitigation (0.06 nM/g Hb) of isoniazid induced increase in MDA content (Figure 8).

### 3.10. Effect of Extracts on Hepatic Markers in Serum

The results of serum biochemical parameters in the control and various experimental groups are depicted in Table 2. Administration of isoniazid in rats by oral route caused liver damage as indicated by a significant increase in serum enzymes ALP, SGOT, SGPT activity, and creatinine, bilirubin contents while decrease in hemoglobin level was observed as compared with control rats (Table 2). Coadministration of rats with S*. podophyllum* and* E. crassipes* extracts with isoniazid accounted for altered levels of serum biochemical markers. Administration of* S. podophyllum* aqueous extract showed elevation in drug induced serum biomarkers indicating liver damage. Liv-52 and* E. crassipes* leaf aqueous extract restored the hepatic marker levels in serum.* E. crassipes* extract (400 mg/kg body weight) restored ALP (69.22%), SGOT (29.91%), SGPT (62.31%), creatinine (108.80%), bilirubin total (48.95%), bilirubin direct (40.22%), and haemoglobin (65.69%) level towards normal values (Table 2).

## 4. Discussion

Plant products have been used for thousands of years in human medicine as therapeutic agents. Bioactivity of phytochemicals has been described extensively in the literature [32]. Natural compounds have been reported to interact with different molecular and cellular targets such as enzymes, transmembrane transporters, hormone, and neurotransmitter receptors [33, 34]. Thus, there are increasing numbers of novel plant species and by-products that are being identified and studied for their potential use in the pharmacological, medical, and agricultural industries [32]. Present work describes the phytochemical characterization, antioxidant, antibacterial, membrane protective activity, and cytotoxic effects of various* S. podophyllum* and* E. crassipes* leaf aqueous extracts. The study also exhibited the effect of test extracts on isoniazid induced oxidative stress and hepatic marker alterations in albino Wistar rats. Phytochemical analysis of aqueous extracts of SP and EC showed presence of flavonoids, terpenoids, reducing sugars, alkaloids, and saponins. Quantitative estimation of phenolics and flavonoid content in* S. podophyllum* and* E. crassipes* extracts demonstrated that flavonoid constitutes the minor part of phenolic compounds in the test fractions. Phenolic compounds have therapeutic potential against different diseases because of their antioxidant property. They are known to possess antispasmodic, antiviral, anti-inflammatory, antisecretory, antiulcer, antidiarrheal, and antitumor activities. Flavonoids are a group of polyphenolic substances present in most plants and are responsible for various biochemical and antimicrobial activities. They exert their antioxidant activity via radical scavenging, metal ion chelation, and membrane protective efficacy [35, 36]. Biological activities observed in the SP and EC extracts might be corroborated with the amount of phenolics. Several studies on phenolic content had been published over the years demonstrating its importance in the medicinal field [36].

In reducing power assay antioxidants act as electron donor which reduces the Fe^3+^ complex to its Fe^2+^. The reducing power of extracts was indicated by higher absorbance values. The experimental data (Figure 2) obtained in the present work showed marked reducing power of extracts at higher concentrations. Thus the reducing activity of extracts could be attributed to the presence of phenolic compounds which might act as reductones. Under stressed conditions iron-containing molecules sequester free iron in the body. Increased levels of iron in the body enhance risk of a variety of cancers [37, 38]. The transition metal ions possess the ability to move single electrons which allows the formation and propagation of many radical reactions. Chelation of metal ion is the main strategy to avoid ROS generation that is associated with redox active metal catalysis [10, 39]. Results have shown that presence of SP and EC leaf extracts in reaction mixture led to decline in formation of Fe^2+^-ferrozine complex indicating the chelation of iron by phytochemicals present in the test plants. Other studies on chelation of iron by plant extracts substantiate our findings. Chelating agents form sigma bonds with metals and are effective as secondary antioxidants. They reduce the redox potential, thereby stabilizing the oxidized form of the metal ion [35, 40–42].

DPPH assay has been largely used as a quick, reliable, and reproducible parameter for screening* in vitro* antioxidant activity of pure compounds as well as plant extracts. Its violet colour is reduced to a yellow coloured product in the presence of antioxidant. The present study revealed the discoloration of reaction mixture suggesting the scavenging potentials of the* S. podophyllum* and* E. crassipes* extracts. DPPH radical scavenging activity of test extract showed the proton donating ability and thereby acting as antioxidant [43]. Higher radical scavenging potential in* E. crassipes* aqueous extract could be attributed to the presence of higher content of phenolic compounds [21].

Metal ion can stimulate lipid peroxidation by the Fenton reaction. Lipid peroxidation causes damage to unsaturated fatty acids, which results in decreased membrane fluidity and leads to many other pathological events. Redox chemistry of iron plays an important role in both the occurrence and the rate of lipid peroxidation. Fe^3+^ reacts with lipid hydroperoxides to form radicals that initiate a chain reaction by reacting with other molecules producing MDA, which is usually taken as a marker of lipid peroxidation (LPO) and oxidative stress [26, 44]. Leaf extract of* S. podophyllum* and* E. crassipes* exhibited considerable lipoprotective efficacy in liver brain and kidney tissue of albino Wistar rats. In* E. crassipes* comparatively higher positive correlation (*r* ^2^ = 0.958–0.978) was found between lipoprotective efficacy and metal ion chelating ability than* S. podophyllum* extract (*r* ^2^ = 0.745–0.767) in different tissues. It may be inferred that phenolics present in the leaf extracts are responsible for quenching metal ion (Fe) and thereby preventing oxidative damage to lipids and thereby providing protection of liver and other tissues [26]. This fact is supported by strong positive correlation between total phenolic content present in test extracts and the biochemical parameters studied (Table 3).

Plants are important sources of naturally occurring antimicrobial and anticancer agents. They are known to possess certain chemicals which are toxic to bacteria and cancer cells. Antimicrobial and cytotoxic activity of plant extracts has also been validated in the literature [36, 45]. Some of these observations have helped in identifying the active principles responsible for such activities and in the development of drugs for the therapeutic use in human beings. Because of emergence of multiple drug resistance in human pathogenic organisms and adverse effects of cancer chemopreventive drugs search for new antimicrobial and anticancer substances from alternative sources including plants is gaining momentum [5, 10]. The plants studied in the current work exhibited substantial antibacterial activities (Table 1) as shown by ZOI (18–22 mm) values of extracts against Gram-negative bacteria* P. vulgaris* (MTCC 7299),* S. typhi* (MTCC 3917), and* B. bronchiseptica* (MTCC 6838). Study revealed that the Gram-positive bacteria exhibited resistance to* S. podophyllum* and* E. crassipes* leaf aqueous extracts. Gram-negative bacteria are frequently reported to have developed multidrug resistance to many of the antibiotics currently available in market [46]. However the current study has demonstrated that phytochemicals present in the leaf aqueous extracts of the test plants have potential to fight Gram-negative bacteria. Low to moderate cytotoxic activity was observed in* S. podophyllum* and* E. crassipes* leaf aqueous extracts. Both extracts showed 73–85% cytotoxic activity against NCI-H322.

Since extracts exhibited considerable* in vitro* antioxidant potential and hence their* in vivo* effect on oxidative stress markers in liver and blood was studied in isoniazid administered albino Wistar rats. Present study proved that* E. crassipes* possesses protective activity against isoniazid induced oxidative stress and hepatotoxicity in rats. Isoniazid has been widely used for the treatment of* Mycobacterium tuberculosis,* but at the same time it is also known to cause hepatotoxicity [47]. It is metabolized to acetylhydrazine and hydrazine by N-acetyltransferase and amidohydrolase. Acetylhydrazine is a toxic metabolite that can covalently bind to liver proteins* in vivo,* but hydrazine is predominantly responsible for isoniazid hepatotoxicity [48]. Hydrazine is known to deplete glutathione in hepatocytes, indicating an elevation in the production of free radicals after hydrazine treatment. Reports suggest that the oxidative stress induced by free radicals results in isoniazid mediated hepatotoxicity [49]. The ferric reducing ability of plasma (FRAP) and measurement of MDA content in RBC ghost were taken as the markers of oxidative stress in the present study. FRAP assay is based on the reduction of Fe^3+^ to Fe^2+^ due to the action of antioxidants. Subsequently, the Fe^2+^ formed may interact with 2,4,6-tris(2pyridyl)-*s*-triazine (TPTZ) providing a strong absorbance at 593 nm [50]. Increased level of MDA in erythrocyte has been reported in many disease conditions which are accompanied with oxidative stress. Increased erythrocyte lipid peroxidation is known to cause a decrease in the fluidity of the membrane lipid bilayer, alteration of integrity, permeability, and functional loss [51]. A decrease in the antioxidant capacity of plasma and increase in MDA content were observed in isoniazid induced oxidative stress in experimental rats.* E. crassipes* leaf aqueous extract supplementation to experimental rats augmented the antioxidant capacity and decreased MDA content which showed the* in vivo* antioxidant potential of extract (Figures 7 and 8). On the other hand* S. podophyllum* leaf extract accounted for toxicity as indicated by enhancement of oxidative stress marker profile in the isoniazid treated rat. Analysis of enzymological and biochemical profile of blood is widely used as indicator to access the functional status of health. Alterations in liver enzyme activities (SGOT, SGPT, and ALP) tend to suggest liver dysfunction in the experimental animal model. Usually an elevation in the liver enzymes may indicate inflammation or damage to the cells in the liver [52]. This results in leakage of higher than normal amounts of certain chemicals (bilirubin and creatinine) and liver enzymes into the blood. The study exhibited elevation in activity of serum enzymes ALP, SGOT, and SGPT as well as bilirubin and creatinine while exhibiting decrease in Hb content in isoniazid induced rats (Table 2). Since these are the diagnostic markers of liver damage and their elevation suggested that isoniazid induces oxidative stress and hepatotoxicity, coadministration of* E. crassipes* leaf AQ extract with the isoniazid was responsible for mitigation of toxicity at biochemical level as indicated by the decrease in toxic markers (hepatic enzymes, creatinine, and bilirubin) along with increase in Hb content. Similar activity was observed with the Liv 52, a well-known hepatoprotectant. On contrary the treatment of* S. podophyllum* leaf AQ extract caused the elevation of hepatic marker enzymes and other parameters in rats indicating the toxic nature of the extract* in vivo*. The study demonstrated the hepatoprotective nature of* E. crassipes* leaf aqueous extract.

## 5. Conclusion

Phytochemicals are large and diverse group of compounds of natural origin. The study established that phytoconstituents present in* E. crassipes* leaf have potent antimicrobial, cytotoxic, antioxidant, and hepatoprotective activity* in vitro* and* in vivo*.* S. podophyllum* also possesses antibacterial and cytotoxic potential.

## Figures and Tables

**Table 1:** Antibacterial activity of *S. podophyllum* and *E. crassipes* leaf aqueous extract.

Bacteria	Zone of inhibition (mm)			
	*S. podophyllum *	*E. crassipes *	Meropenem	Ampicillin
*Bacillus cereus* (MTCC 6840)	10	08	16	15
*Streptococcus mutans* (MTCC 497)	08	10	08	08
*Proteus vulgaris* (MTCC 7299)	22	20	08	15
*Salmonella typhi* (MTCC 3917)	22	22	15	15
*Bordetella bronchiseptica* (MTCC 6838)	18	08	08	08

Zone of inhibition (ZOI) is shown as average of three replicates.

**Table 2:** Effect of *S. podophyllum* and *E. crassipes* leaf aqueous extracts on isoniazid induced alterations of serum markers in Wistar rats.

Parameters	Control	INH	INH + L52	INH + SP	INH + EC
CRE mg/dL	0.353 ± 0.11	1.830 ± 0.06∗∗∗	0.550 ± 0.19^#^	1.800 ± 0.06∗∗∗	0.223 ± 0.10^#^
BIL total mg/dL	0.247 ± 0.06	0.580 ± 0.05∗∗	0.300 ± 0.07^#^	0.660 ± 0.10∗∗	0.417 ± 0.14^#^
BIL direct mg/dL	0.113 ± 0.03	0.297 ± 0.06∗	0.153 ± 0.03^#^	0.433 ± 0.10∗∗∗	0.223 ± 0.10^#^
ALP U/L	29.85 ± 7.90	220.64 ± 45.39∗∗∗	47.63 ± 10.37^#^	246.25 ± 47.19∗∗∗	88.57 ± 23.59^#^
SGOT U/L	40.66 ± 11.55	174.95 ± 19.80∗∗∗	50.45 ± 4.37^#^	184.74 ± 19.10∗∗∗	131.78 ± 18.57^#^
SGPT U/L	38.73 ± 6.91	138.24 ± 10.96∗∗∗	49.67 ± 5.74^#^	148.43 ± 10.94∗∗∗	76.24 ± 10.68∗∗
Hb mg/dL	13.35 ± 0.66	10.96 ± 1.86^#^	12.21 ± 1.33∗∗∗	6.88 ± 0.38^#^	11.78 ± 0.25^#^

Data represent mean ± SD of three replicates (*P* < 0.05). One-way ANOVA followed by Dunnett's Multiple Comparison Test was used to compare control group from other experimental groups (^#^ *P* < 0.05, *P < 0.01, **P < 0.001, and ***P < 0.0001). INH: isoniazid, L52: Liv52, SP: *Syngonium podophyllum*, EC: *Eichhornia crassipes*, CRE: creatinine, BIL: bilirubin, ALP: alkaline phosphatase, SGOT: serum glutamic oxaloacetic transaminase, SGPT: serum glutamate pyruvate transaminase, and Hb: haemoglobin.

**Table 3:** Relationship between total phenolic content and biochemical activities of *S. podophyllum* and *E. crassipes* leaf aqueous extracts.

Extract	RP	DPPH	MIC	LPOI		
				Liver	Brain	Kidney
SP	(+)/*r* ^2^ = 0.976	(+)/*r* ^2^ = 0.893	(+)/*r* ^2^ = 0.954	(+)/*r* ^2^ = 0.930	(+)/*r* ^2^ = 0.834	(+)/*r* ^2^ = 0.938
EC	(+)/*r* ^2^ = 0.983	(+)/*r* ^2^ = 0.834	(+)/*r* ^2^ = 0.915	(+)/*r* ^2^ = 0.988	(+)/*r* ^2^ = 0.992	(+)/*r* ^2^ = 0.985

Sign in parentheses indicates positive (+) correlation. RP: reducing power, DPPH: radical scavenging activity, MIC: metal ion chelating activity, LPOI: lipid peroxidation inhibition, SP: *Syngonium podophyllum*, EC: *Eichhornia crassipes*, and *r* ^2^: correlation regression coefficient.
